# Phosphorylation-dependent assembly of DNA damage response systems and the central roles of TOPBP1

**DOI:** 10.1016/j.dnarep.2021.103232

**Published:** 2021-12

**Authors:** Matthew Day, Antony W. Oliver, Laurence H. Pearl

**Affiliations:** a Cancer Research UK DNA Repair Enzymes Group, Genome Damage and Stability Centre, School of Life Sciences, University of Sussex, Falmer, Brighton BN1 9RQ, UK; b Division of Structural Biology, Institute of Cancer Research, Chester Beatty Laboratories, 237 Fulham Road, London SW1E 6BT, UK

**Keywords:** BRCT domains, Phosphopeptide binding, Specificity, Protein-protein interactions, DNA damage response, Checkpoints

## Abstract

The cellular response to DNA damage (DDR) that causes replication collapse and/or DNA double strand breaks, is characterised by a massive change in the post-translational modifications (PTM) of hundreds of proteins involved in the detection and repair of DNA damage, and the communication of the state of damage to the cellular systems that regulate replication and cell division. A substantial proportion of these PTMs involve targeted phosphorylation, which among other effects, promotes the formation of multiprotein complexes through the specific binding of phosphorylated motifs on one protein, by specialised domains on other proteins. Understanding the nature of these phosphorylation mediated interactions allows definition of the pathways and networks that coordinate the DDR, and helps identify new targets for therapeutic intervention that may be of benefit in the treatment of cancer, where DDR plays a key role. In this review we summarise the present understanding of how phosphorylated motifs are recognised by BRCT domains, which occur in many DDR proteins. We particularly focus on TOPBP1 – a multi-BRCT domain scaffold protein with essential roles in replication and the repair and signalling of DNA damage.

## 1 Introduction

The DNA Damage Response (DDR) is an extraordinary, transient reconfiguring of cellular behaviour, driven by a massive cascade of post-translational events (primarily phosphorylation) downstream of DNA damage detection. Even in a simple model organism such as budding yeast – *S. cerevisiae* – exposure to DNA damaging agents results in substantially increased phosphorylation of more than 1500 sites across hundreds of proteins [1]. The mechanistic role of only a tiny fraction of these phosphorylation events has been studied.

Here, we review the present understanding of how this phosphorylation facilitates assembly of the large multiprotein complexes that allow cells to orchestrate their response to DNA damage and to maintain genome stability. In particular we focus on the function of BRCA1 C-terminus (BRCT) domains in recognition of phosphorylated motifs in DDR proteins, and the role of DNA topoisomerase II binding protein 1 (TOPBP1) – a large scaffold protein containing multiple BRCT domains - that plays key roles in regulating replication, DNA break repair and mitosis.

## 2 γ-H2AX and BRCT domains

Since its discovery [2], phosphorylation of the most C-terminal serine residue (Ser 139) in the histone H2A variant H2AX has become the definitive biomarker for the presence of DNA double-strand breaks (DSBs) in mammalian cells [3]. Comparable phosphorylations of closely related C-terminal motifs are also observed in both of the yeast species commonly used in genome stability studies, *Saccharomyces cerevisiae* [4] and *Schizosaccharomyces pombe* [5] but here the phosphorylation occurs on the normal histone H2A isoform rather than a specialised variant.

**Fig. 1:**
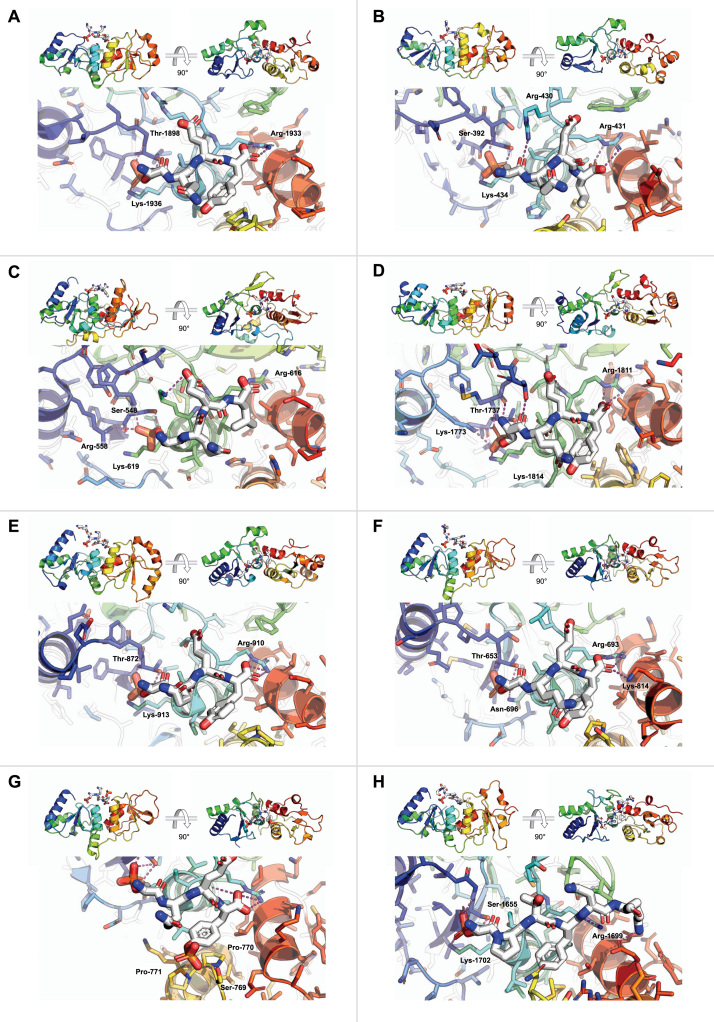
**Phosphopeptide bound structures of the γH2AX or γH2A C-terminal BRCT**_**2**_**modules.** BRCT domain secondary structure cartoons are rainbow-coloured (N-terminus blue - > C terminus red), the histone tail peptides shown as white sticks, and with dashed magenta lines indicating hydrogen bonding interactions between the chains. (**A**)MDC1 (PDB: 2AZM) [8] the phosphate of γH2AX peptide is coordinated by Thr-1898 and Lys-1936 of MDC1 with the carboxyl-terminus of the peptide recognised by Arg-1933. (**B**)Mdb1 (PDB: 7P0L) (unpublished data) shows a similar arrangement as for the human ortholog with Ser-392 and Lys-434 and Arg-431 taking the role of previously labelled residues. (**C**)Crb2 (PDB: 2VXC) [16] as described in the text. (**D**)53BP1 (PDB: 5ECG) [11] Thr-1737, Lys-1773 and Lys-1814 forming the PB-motif triplet and Arg-1811 interacting with the carboxyl-terminus of the peptide. (**E**)PTIP (PDB: 3SQD) [20] Similarly to MDC1 there is no coordinating residue in the Arginine position of the PB-motif triplet but the other two residues Thr-872 and Lys-913 coordinate the phosphate group with Arg-910 again recognising the C-terminus of H2AX. (**F**)MCPH1 (PDB: 3SZM) [24] Thr653 and Asn-696 take the two crucial phosphate coordinating positions and the carboxylate of Tyr-143 is picked up by Arg-693 and in this case an additional H-bond to Lys-814. (**G**)MCPH1 bound to H2AX pS139 pY142 peptide (PDB: 3U3Z) [25] Ser-769 in the BRCT domain forms a H-bond with the phosphate group of pTyr-142, while the sidechain of H2AX Gln-140 is altered to accommodate the phosphate, but the positioning is otherwise very similar to that of the singly phosphorylated peptide structure. (**H**)BRCA1 bound to FANCJ/BACH1 pS990 (PDB: 1T15) [34] with the phosphorylation picked up by Ser-1655 and Lys-1702 in the BRCT domain. Arg-1699 sits in the equivalent location of the carboxyl coordinating arginine in the histone tail binders, but in this case it picks up a H-bond to the mainchain carbonyl of the residue in the + 3 position.

It was recognised early on that foci of phosphorylated H2AX (known as γH2AX) observed by immunohistochemistry, within the nuclei of cells subjected to genotoxic insult, co-localised with foci of important DNA repair factors such as RAD50, RAD51 and BRCA1 [6]. Based on these observations, it was suggested that the γH2AX modification might function to facilitate decompaction of chromatin, thereby contributing to the indirect recruitment of downstream factors by providing access to the site of damage. However, subsequent studies demonstrated that γH2AX is a direct participant in recruitment of repair factors, and interacts directly with MDC1 [7]; via specific binding of its C-terminal tetrapeptide incorporating pSer139, to the tandem BRCT domain at the C-terminus of MDC1 [8] (Fig. 1**A**). This, and the earlier demonstrations of phosphorylation-dependent interactions of BRCA1 and PTIP [9], [10], highlighted the key role played by BRCT domains in mediating interactions between proteins involved in the DNA damage response.

The high affinity interaction between γH2AX and the tandem BRCT domain pair (BRCT_2_) module at the C-terminus of MDC1 (0.4 μM, in vitro) [11] is a key step in the amplification and spreading of the γH2AX signal via recruitment of MRN and ATM, which in metazoan cells also facilitates recruitment of 53BP1 and the associated Shieldin complex to DNA double strand breaks, through ubiquitylation of histone H2A [12], [13]. Although no MDC1 homologue is evident in *S.cerevisiae*, Mdb1 in *S.pombe* has a similar molecular architecture and shares the ability of MDC1 to interact with the equivalent γH2A modification via its C-terminal BRCT_2_ module [14] (Fig. 1**B**). However, unlike MDC1, which is essential for genomic stability, Mdb1 appears to be largely dispensible.

The central role of MDC1 in metazoan DSB repair, has tended to dominate thinking about the function of γH2AX as a DNA damage signal, and understanding of its recognition by other factors has been less well explored.

### 2.1 C-terminal BRCT_2_ modules

A C-terminal BRCT_2_ module also occurs at the C-terminus of *S.cerevisiae* Rad9p (not to be confused with *S.pombe* and metazoan RAD9 which is a part of the 9–1–1 complex), *S.pombe* Crb2, and metazoan 53BP1. The BRCT_2_ modules in both Rad9p and Crb2 have been shown to interact with γH2A, and mutational disruption of this interaction impacts DNA damage responses [5], [15]. Structural analysis of the Crb2-BRCT_2_ module revealed a similar mode of interaction with the C-terminal phosphopeptide motif of γH2A as that seen in the interaction of MDC1-BRCT_2_ with MDC1, allowing the identification of key features in the BRCT_2_ module that mediate the specificity for the phosphorylated serine and carboxyl-terminus [16]. Critical to the ability of BRCT domains to recognise the phosphorylated status of ligand peptides, are a triplet of amino acids (Ser548, Arg558 and Lys619 in Crb2) which make multiple hydrogen binding and charge-neutralising interactions with the negatively-charged phosphate group of the phosphorylated peptide (Fig. 1**C**). The presence of this phospho-binding (PB) triplet motif is fully diagnostic of the ability of a BRCT domain to interact with phosphorylated sites on ligand proteins.

Despite the strong conservation of these features in 53BP1, a number of studies suggested that its BRCT_2_ module was largely dispensable for its recruitment to sites of damage and its function in DSB repair [17], [18]. However, subsequent structural and biochemical studies showed a direct and specific interaction of 53BP1-BRCT_2_ with γH2AX (Fig. 1**D**), and demonstrated DNA repair defects when this interaction was disrupted in cells, that particularly impacted on the late-phase repair of persistent DSBs in heterochromatin and other highly condensed chromatin settings [11], [19].

Metazoan PTIP, *S.cerevisiae* Rtt107 and *S.pombe* Brc1 have also been shown to bind to γH2AX in a similar manner to MDC1 and 53BP1 [20] (Fig. 1**E**), or γH2A in the case of Rtt107 [21] and Brc1 [22]. Of the other metazoan proteins that contain a C-terminal BRCT domain, Microcephalin (MCPH1) is the only other known to be a γH2AX interacting partner [23] and it has no known equivalent in yeast. Despite the PB-motif in the C-terminal BRCT_2_ module containing an Asn in the place of the conserved Lys residue it is able to coordinate phosphorylated ligands and a crystal structure of the complex demonstrated the expected conformation [24] (Fig. 1**F**).

The structure of the C-terminal BRCT_2_ module of MCPH1 bound to a H2AX peptide where Tyr-142 is phosphorylated in addition to Ser-139, has also been determined [25] (Fig. 1**G**). Tyr-142 is constitutively phosphorylated by WTSF and has been shown to modulate the DNA damage response, being gradually dephosphorylated by the Eyes Absent phosphatases [26] as it progresses [27], while also being responsible for the recruitment of pro-apoptotic factors [28]. MCPH1 can clearly accommodate the phosphorylated tyrosine modification, and while it is currently unclear what affect it would have on the interaction with PTIP, it has been demonstrated that both MDC1 [29] and 53BP1 [11] discriminate against binding to a pS139 pY142 H2AX peptide. A number of mass spectrometry studies have been unable to identify the pY142 modification in vivo [30], [31], creating doubt about how widespread the existence of this marker is, however it is clear that where found it would enable differentiation of which proteins were able to bind to γH2AX.

While the C-terminal BRCT_2_ modules of MDC1, 53BP1, PTIP and MCPH1 all appear to be specifically adapted for unique recognition of the highly acidic terminal phosphorylated-motif of γH2AX, the structurally homologous BRCT_2_ module at the C-terminus of BRCA1 is able interact more promiscuously with phosphorylation sites embedded within their ligand proteins’ amino acid sequence, that incorporate a pSer-X-X-Phe motif [32]. Structurally characterised ligands for BRCA1-BRCT_2_ include other DNA repair factors such as FANCJ/BACH1 [33], [34] (Fig. 1**H**), BARD1 [35], CtIP [36], ATRIP [37], BRAT1/BAAT1 [37] and Abraxas [38], but also a metabolic enzyme Acetyl-CoA Carboxylase [39]. Other phospho-dependant ligands of BRCA1-BRCT_2_ are likely to be identified.

### 2.2 MCPH1 and PTIP – alternative BRCT domain arrangements

In addition to the C-terminal pair, MCPH1 contains an additional singleton BRCT domain at its N-terminus that lacks the PB-motif residues [40]. This BRCT domain has been shown to interact in a non-phosphorylation dependent manner with a number of other proteins including SET [41] and the BAF170 component of the SWI/SNF chromatin-remodelling complex [42]. Although the exact structural basis of the interactions made by this singleton BRCT domain remain to be determined, mutations made in a hydrophobic pocket located in the corresponding position to the phosphate binding pocket, prevent the ability of MCPH1 to rescue phenotypes caused by a MCPH1 deletion [40], demonstrating that BRCT domains have evolved to be capable of distinct interaction modes.

PTIP and the related yeast proteins Brc1 and Rtt1107 each consist of a tandem arrangement of three BRCT-pairs including a C-terminal BRCT_2_ module that interacts with γH2AX (BRCTs 5 and 6).

**Fig. 2:**
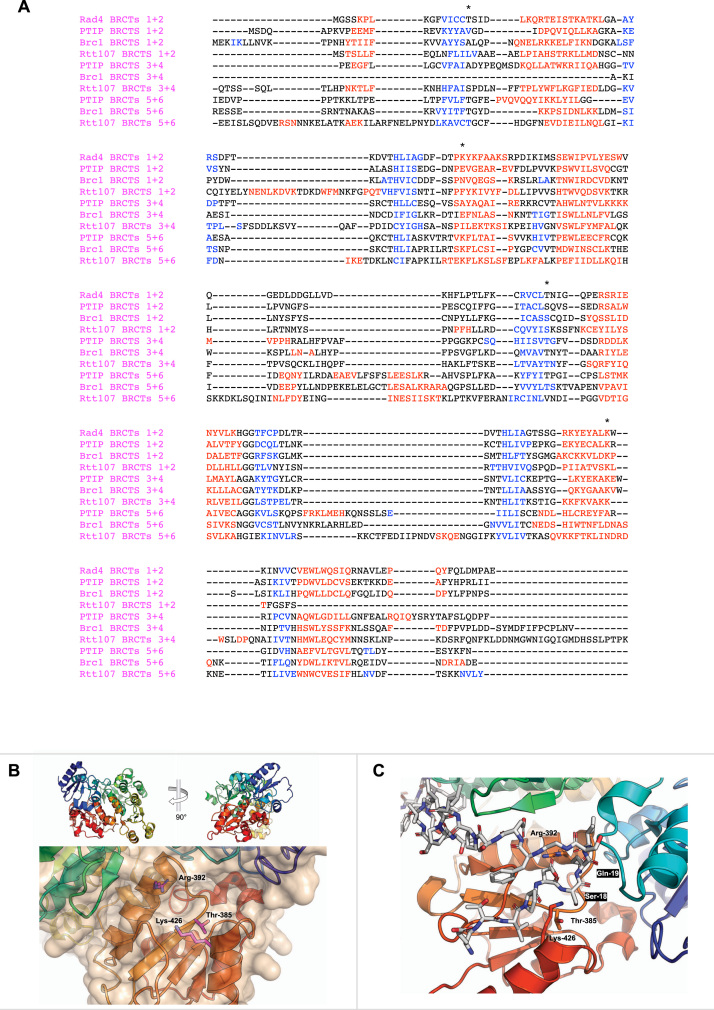
**PB-motifs in PTIP, Brc1 and Rtt107 BRCT domains.** (**A**) Structure based alignment of PTIP, Brc1 and Rtt107 BRCT domain pairs, including RAD4 BRCTs 1 and 2 as both have PB motif, using PROMALS3D Webserver. Helices coloured red, strands in blue, and PB-motif residues are marked with an asterix. PB-motifs can be seen in PTIP BRCTs 2, 4 and 5, Brc1 BRCTs 4 and 5 and Rtt107 BRCTs 4 and 5. (**B**) BRCT domain secondary structure cartoon representation of BRCTs 1–4 of Rtt107 (PDB 6J0V) rainbow-coloured (N-terminus blue - > C terminus red), with the PB-motif residues highlighted as magenta sticks, and the surface represented showing the groove on the surface. (**C**) Nse6 bound structure of Rtt107 (PDB 6J0W) showing the proximity of Serine-18 in Nse6 to the phosphate binding site in BRCT 4 of Rtt107. The Nse6 peptide is shown as white sticks and labelled with white lettering on black background.

In PTIP, both other BRCT pairs contain a single PB motif, however these are found in the second BRCT of each module (BRCTs 2 and 4) distinguishing them from the canonical BRCT_2_ arrangement (Fig. 2**A**). While there are no structures demonstrating phosphorylation dependent interactions, a number of candidates have been identified in the literature. A region corresponding to BRCTs 3,4,5 and 6 of PTIP is required for focus formation [43] and when Serine-25 in 53BP1 is phosphorylated it is able to interact with PTIP in a way that requires both C-terminal pairs of BRCT domains in PTIP [44]. Close examination of the data in these papers suggests some recruitment for a construct without the C-terminal pair, but all four being required for a robust recruitment or interaction, placing Ser-25 of 53BP1 as a candidate for interaction with BRCT4 of PTIP. Artemis has been clearly identified as interacting with BRCT2 of PTIP promoting the use of the NHEJ repair pathway over HR [45].

For the two yeast proteins, the only other PB-motif is found in BRCT4. While no ligands have yet been identified for Brc1, pThr-80 of histone H4 has been shown to interact with BRCT 4 of Rtt107 with the interaction being important for inactivation of the signalling cascades that follow the DNA damage checkpoint [46].

The N-terminal BRCT-pair of Rtt107 lacks a conserved PB motif, however it has been shown to form an unusual ‘tetra-BRCT’ arrangement, involving BRCTs 1, 2, 3 and 4, which is able to scaffold non-phosphorylation dependent interactions with various DNA repair factors using a region located between the binding pockets of BRCTs 2 and 4 [47]. The arrangement of this module is such that a channel exists between BRCTs 1 and 4 that could accommodate a phosphorylated motif binding into the PB motif of BRCT4 (Fig. 2**B)**, although given the proximity to the binding site for the phosphorylation independent interactions seen in the crystal structures, the two types of interaction would likely exclude each other. In fact, in the Nse6 bound structure, serine-18 of the peptide, which is followed by a glutamine making it a consensus Mec1/Tel1 site, sits tantalisingly close to the PB in BRCT 4 (Fig. 2**C**) and it would be interesting to see if the interaction is enhanced by its phosphorylation. The overall architecture of PTIP is different to the yeasts due to a large insertion, including a polyQ tract, between BRCT domains 2 and 3 and therefore it is unclear as to whether it would be able to form the same tetra-BRCT arrangement.

Detailed molecular dissection of PTIP interactions is in its infancy and clearly more work is required to enable an understanding of the different phosphorylation dependent complexes it scaffolds.

## 3 TOPBP1/Rad4/Dpb11 – an abundance of BRCTs

Although the BRCT domain is named in recognition of its presence at the C-terminus of BRCA1 [48], its occurrence as a repeated functional module was first noted in the product of the *S.pombe* radiation-sensitivity gene Rad4 [49], [50], [51], which contains four copies arranged as two tandem pairs. A similar architecture is possessed by the *S.cerevisiae* homologue Dpb11 [52]. Cloning of the human orthologue of Rad4 and Dpb11 - TOPBP1 - revealed a more elaborate architecture in which eight BRCT domains could be identified [53]. Comparison of the mammalian and yeast sequences suggest that the larger structure of TOPBP1 is not a simple duplication of the smaller yeast arrangements, but instead results from a complex pattern of evolutionary modifications, with the insertion of a singleton BRCT domain, BRCT3, between the tandem pairs that are homologous to those of the yeast proteins, and the addition of a further singleton, BRCT6, and an additional tandem pair at the C-terminus [54]. Structural analysis of this C-terminal tandem pair BRCT7,8 of human TOPBP1 [55], revealed a very similar architecture to the ‘canonical’ BRCT_2_ structures in MDC1, 53BP1, BRCA1 and their yeast homologues, and TOPBP1-BRCT7,8 shares the ability of BRCA1-BRCT_2_ to interact with the DNA helicase FANCJ/BACH1 in a phosphorylation-dependent manner, albeit through a different phosphorylated motif [56].

Structural analysis of the N-terminal BRCT repeats of TOPBP1 and Rad4, revealed a very different arrangement of the BRCT domains to that seen in the canonical BRCT_2_ structures [57], [58], [59]. Most surprising was the presence of an additional BRCT domain – designated BRCT0 – at the N-terminus of TOPBP1, which is absent from the yeast Rad4. Unlike the canonical BRCT_2_ modules in which only the first domain carries the PB-motif and the two BRCT domains combine to offer a single phosphopeptide binding site, the PB-motif was present in both BRCT1 and BRCT2 in TOPBP1/Rad4, suggesting that they might each be able to bind a phosphopeptide ligand independently. BRCT0 which is only present in TOPBP1 lacks the PB-motif and its biological function remains obscure. The central BRCT-pairs of TOPBP1 (BRCT4,5) and Rad4 (BRCT3,4) have an arrangement of their component domains that is distinct from both the canonical modules and the TOPBP1/Rad4 N-terminal modules [59], [60]. Like the canonical BRCT_2_ modules, only one BRCT domain contains a PB-motif, but in this case, it is in the second domain – BRCT5 in TOPBP1, BRCT4 in Rad4. Neither of the singleton BRCT domains in TOPBP1 have PB-motifs.

### 3.1 Scaffolding enzymes

**Fig. 3:**
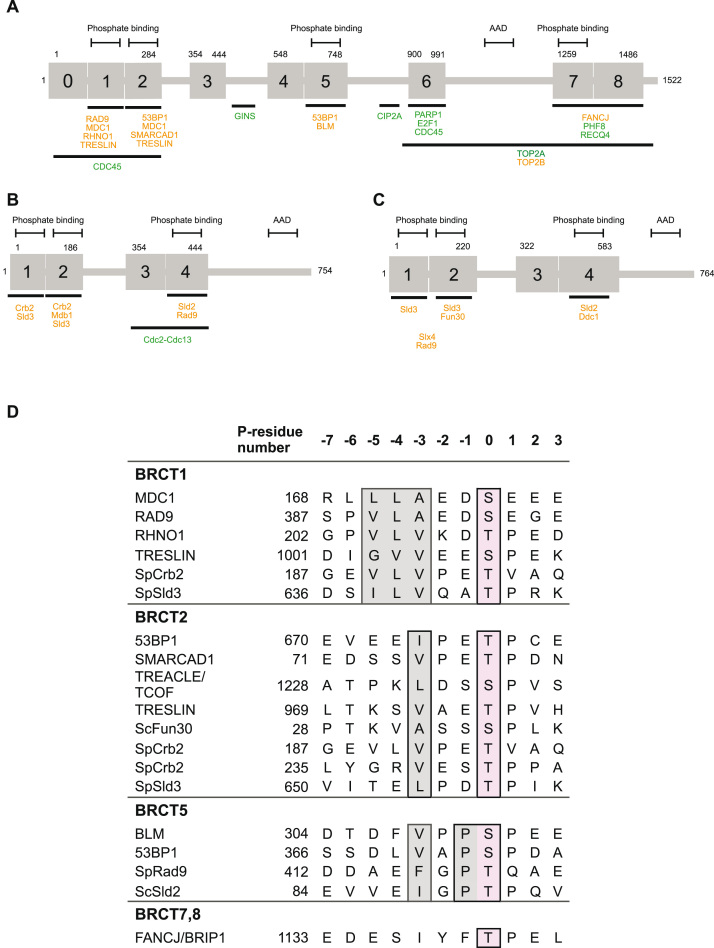
**Mapped interactions of TOPBP1, Rad4 and Dpb11.** Phosphorylation dependent, and phosphorylation independent, interactions are shown in orange and green respectively. (**A**) Schematic representation of human TOPBP1 showing interactions with RAD9 [58], MDC1 [81], RHNO1 [80], 53BP1 [77], SMARCAD1 [61], TRESLIN [80], GINS [115], BLM [63], CIP2A [116], PARP1 [65], E2F1 [71], CDC45 [95], FANCJ/BACH1 [55], PHF8 [68], RECQL4 [99], TOP2A [64], TOP2B [53]. (**B**) Schematic representation of *S.pombe* Rad4 showing interactions with Mdb1 [80], Crb2 [59], Sld3 [94], Rad9 [109], Sld2 [94], Cdc2-Cdc13 [59]. (**C**) Schematic representation of *S.cerevisiae* Dpb11 showing interactions with Rad9 [117], Slx4 [118], Fun30 [61], Sld3 [92], Ddc1 [118], Sld2 [97]. (**D**)Alignment of experimentally determined phosphorylated motifs binding to different BRCT domains of TOPBP1. The residue number indicates the phosphorylated residue. The key specificity determinants for binding to each site are highlighted.

A range of ligand proteins for TOPBP1 and/or its orthologues, have been identified, whose interactions are mediated through binding of a phosphorylation site (or sites) on the ligand protein, to one or more of the PB-motif containing BRCT domains of TOPBP1 (Fig. 3**A-C)**. Several of these are ATP-dependent DNA helicases whose ability to interact with TOPBP1 is critical to their biological function: the chromatin remodeller SMARCAD1 and its *S.cerevisiae* orthologue FUN30, interact with the N-terminal BRCT1,2 module of TOPBP1 and Dbp11 respectively [61]; the RECQ-family helicase BLM interacts with the BRCT4,5 module of TOPBP1 [62], [63]; and FANCJ/BACH1, a helicase involved in alleviation of replication stress, binds to the C-terminal BRCT7,8 module [55], [56]. It is currently unclear whether association with TOPBP1 plays a direct mechanistic role in the function of these helicases, or just serves to localise them to their sites of action.

TOPBP1 also interacts with both of the type-II DNA topoisomerases, TOP2A [64] and TOP2B [53] – the latter of which gives TOPBP1 its name. In both cases a C-terminal segment of TOPBP1 including BRCT6 and the canonical phosphopeptide-binding BRCT7,8 module is required for the interaction, and at least for TOP2B there is indirect evidence that the interaction is mediated by phosphorylation, although the site (or sites) involved have not been identified. As with the TOPBP1 helicases, the main function of the interaction is to localise the topoisomerase to a site of action on the genome – ultrafine anaphase bridges (UFBs) in the case of TOP2A – and abrogation of the interaction results in a failure to resolve these structures [64]. Interestingly, the same study identified a requirement for the PB-motif of TOPBP1-BRCT5 to recruit TOPBP1 itself to UFBs, implying a requirement for interaction with a phosphorylated motif of a protein earlier in the recruitment cascade. However, none of the known BRCT5 phospho-dependent interactors fulfilled this role, and the identity of this protein remains unknown.

Interaction with PARP1 is believed to be mediated through BRCT6 of TOPBP1 [65]. It was initially suggested this may be achieved through a conserved PAR binding motif in BRCT6, however structural elucidation revealed the motif to be buried, ruling out that interaction mode [66]. The use of in vitro translated proteins in this study, along with the lack of the PB-motif residues in BRCT6, would suggest the interaction is unlikely to be phosphorylation dependent.

Recently, the Jmjc-domain lysine demethylase PHF8 was identified as a TOPBP1 ligand protein, with a casein kinase 2 (CK2)-phosphorylated motif at Ser 854 in PHF8, apparently binding to the canonical BRCT7,8 module of TOPBP1 [67]. However, this is called into question by recent data [68] that structurally characterises an unusual phosphorylation-independent interaction of the region around Ser 854 with BRCT7,8 and suggests a role for PHF8 in facilitating TOPBP1 interactions through targeted demethylation of Lys118 within the PB-motif of BRCT1. Clearly further work will be required to fully delineate the role of PHF8 in the complex functions of TOPBP1.

Although not an enzyme itself, but rather a regulator of one - the protein phosphatase PP2A inhibitor CIP2A has recently been characterised as an essential TOPBP1 interactor [69], [70]. CIP2A appears to primarily interact with a region of TOPBP1 between BRCT5 and BRCT6, but whether its interaction is dependent on phosphorylation is unclear. The two studies make different conclusions about the function of CIP2A in TOPBP1 biology, with one [69] showing a particular involvement in the repair of DNA double-strand breaks occurring during mitosis, while the other [70] suggesting a more general role in dampening DNA damage checkpoint signalling. In any event, CIP2A appears to be an essential factor for the roles of TOPBP1 in maintaining genomic stability, but more work will be required to understand the biochemical mechanism by which this is accomplished.

A direct interaction between BRCT6 of TOPBP1 and the transcription factor E2F1 has been identified [71] although the apparent requirement for phosphorylation [72], despite lack of the PB-motif phosphorylation coordinating residues in BRCT6 [66], would suggest a unique, and as yet unresolved, interaction mode if true.

### 3.2 Scaffold crosstalk

The greatest level of complexity in the assembly of DNA damage response complexes, arises from the phosphorylation-dependent interaction of many of the large scaffold proteins with themselves and each other, generating the extensive linked protein networks that most likely underpin the architecture of the macromolecular assemblies that become visible as DNA damage foci.

TOPBP1 has been shown to associate with all three of the main DDR scaffolds that possess C-terminal BRCT_2_ modules, BRCA1, 53BP1, MDC1 [73], [74], [75]. Of these, the interaction with BRCA1 is the least well characterised, with no direct biochemical interaction having been demonstrated. As both TOPBP1 and BRCA1 interact with FANCJ/BACH1 [76] in a phosphorylation-dependent manner, it is possible that their interaction is indirect, but nonetheless intimate.

In contrast, the interaction of TOPBP1 and 53BP1 is direct and involves simultaneous binding of two phosphorylated 53BP1 motifs centred at Ser 366 and Thr 670 with TOPBP1-BRCT5 and BRCT2 respectively [77], and disruption of these interactions in cells generates a defect in the G1/S checkpoint response. Although the sites contain SP and TP motifs that are often targets of cell-cycle regulatory cyclin-dependent kinases (CDKs), their formation is DNA damage dependent and resistant to CDK inhibitors. The kinase (or kinases) responsible remain unknown. A more complex interaction occurs between the *S.pombe* orthologues of TOPBP1 and 53BP1, Rad4 and Crb2 [78] and is critical for an effective DNA damage response. In this system two canonical CDK phosphorylation sites in Crb2, Thr215 and Thr235, recruit Rad4 through their cooperative interaction with BRCT1 and BRCT2 respectively. This then facilitates phosphorylation of a non-canonical site at Thr187 by the cyclin-dependent kinase Cdc2-Cdc13 which associates with Rad4 via a phosphorylation-independent interaction with the BRCT3,4 module. A single Rad4 molecule then bridges the resulting two phosphorylated Thr187 residues of a Crb2 dimer through their high-affinity interaction with BRCT1 and BRCT2 [59].

Association of MDC1 with TOPBP1 was initially identified as a functionally important contributor to recruitment of TOPBP1 to stalled replication forks and ATR-dependent activation of CHK1 [75]. This association was attributed to interaction of the highly phosphorylated pSDT repeats of MDC1 with BRCT4,5, and was supported by a crystal structure of a consensus pSDT motif bound to BRCT5 [60]. The reported binding mode of this motif was unusual, and in contrast to that observed for BRCT4,5 binding to phosphopeptides from 53BP1 and BLM [63], [77], as it did not involve the phosphate on the phosphorylated serine making the expected set of interactions with the conserved PB-motif. Surprisingly, despite strong conservation on both sides, no interaction was observed between the single SDT motif present in Mdb1, the *S.pombe* homologue of MDC1, and any part of Rad4, the *S.pombe* homologue of TOPBP1. Instead, a high-affinity interaction was found between a known CDK phosphorylation site at Thr113 [79] and BRCT2 of Rad4 [80]. A subsequent analysis of human MDC1 showed no interaction in vitro between any of the six pSDT motifs and TOPBP1 in contradiction to [75], but instead identified two CK2 phosphorylation sites at Ser168 and Ser196, which mediated a functionally significant interaction of MDC1 and TOPBP1 required for stabilisation of DSBs in mitotic cells through the formation of inter-chromosomal bridges [81].

Treacle/TCOF1 is a component of a large ribonuclear protein complex located in the nucleolus, that plays a role in regulating ribosomal RNA transcription in response to DNA damage [82], [83]. Its low level of sequence complexity, lack of identifiable globular regions and high level of posttranslational modification implicate it as a scaffold protein. Like MDC1, treacle/TCOF1 has a repeated array of phosphorylated Ser-x-Thr motifs, which also interact with the FHA domain of NBS1. Treacle/TCOF1 also interacts with TOPBP1 through a cluster of phosphorylation sites that appear to interact with both of the BRCT0,1,2 and BRCT4,5 modules [84], however which phosphorylated residues interact with which BRCT domain has not yet been determined. Recruitment of TOPBP1 by Treacle/TCOF1 facilitates activation of ATR signalling which is required for the nucleolar response to DNA damage.

Several studies have suggested that TOPBP1, like its scaffold partners 53BP1 and MDC1 [85], [86], functions in an oligomeric or at least dimeric state in some of its roles [81], [87], [88], [89]. TOPBP1 certainly forms distinct nuclear foci following DNA damage [77] and recent studies suggest that these may be substantial condensates [90]. However, the formation and distribution of these focal condensates can be very substantially altered by single point mutations in TOPBP1 or in its interacting partner proteins. This suggests that these nano-scale TOPBP1 assemblies result from extensive cross-linked networks of highly specific protein-protein interactions, rather than gross chemical properties of the molecules involved, as would be the case in *bona fide* liquid phase separations. Detailed structural studies will be required to determine the nature and role of homotypic TOPBP1 interactions in their different functional contexts.

### 3.3 Assembling replication complexes

TOPBP1, Dpb11 and Rad4, play key roles in the assembly of the replicative DNA helicase CMG complex and its specific loading at replication origins, through their phosphorylation-dependent interaction with the assembly factors Sld3 and Sld2/Drc1 in budding and fission yeast, and their functional equivalents treslin and RECQL4 in animal cells [91], [92], [93], [94]. Like the interaction of Crb2 with Rad4 [59], interaction of Sld3/treslin involves simultaneous binding of a pair of CDK-phosphorylated sites on the assembly factor, to the BRCT1,2 module of TOPBP1/Rad4/Dpb11 [80].

TOPBP1 BRCT0,1,2, along with BRCT6, have also been shown to interact directly with CDC45 as part of replication initiation [95], although no specific phosphorylation sites on CDC45, or the requirement for phosphorylation, have yet been identified for this interaction. Similarly, the yeast ortholog Dpb11 interacts directly with Cdc45 [96].

The yeast Sld2/Drc1 proteins interact with the BRCT3,4 module of Dpb11/Rad4 via a single CDK phosphorylation site in the N-terminal end of the assembly factor [97], so that Sld2 and Sld3, and their respective binding partners GINS-Polε and Cdc45 are brought together through simultaneous interaction with the same TOPBP1/Rad4/Dpb11 molecule (but see below). Although no mammalian Sld2 homologue has been identified, the RECQ-family helicase RECQL4 has been suggested to fulfil a similar role through its involvement in DNA replication initiation, and the presence of an N-terminal domain with some homology to the N-terminal region of Sld2 implicated in interaction with Dpb11/Rad4 [98]. However, the CDK phosphorylation site that is highly conserved in the yeast proteins is absent from RECQL4, and interaction studies suggest that the N-terminal region of RECQL4 binds in a phosphorylation-independent manner to a C-terminal segment of TOPBP1 including the BRCT7,8 module and an upstream proline-rich region [99].

### 3.4 Assembling checkpoint signalling complexes

Perhaps the best described role of TOPBP1, conserved across Dpb11/Rad4, is as the bridge between the RAD9-RAD1-HUS1 (9−1−1) clamp, and ATR-ATRIP bound via RPA to segments of ssDNA [100], [101], [102]. While the interaction with ATR is mediated by a predominantly unstructured ATR-activating domain (AAD) near the C-terminus of TOPBP1/Dpb11/Rad4 [103], [104], [105], the link to 9–1–1 is mediated by binding of the phosphorylated C-terminus of RAD9/Ddc1/Rad9 [101], [106], [107] to one or other of the upstream BRCT domains. In animals, where the C-terminal phosphorylation of RAD9 in 9–1–1 is constitutive and performed by CK2 [108] binding is highly specific for TOPBP1-BRCT1 [58], [80], whereas in S*.pombe*, where the C-terminal phosphorylation of the Rad9 tail is DNA-damage dependent [109], the interaction is with Rad4-BRCT4 [59].

In animals, the interaction of 9–1–1 and TOPBP1 can also be bridged independently of RAD9 phosphorylation, by RHNO1 [110], a largely unstructured protein that binds to the edge of the 9–1–1 ring via a region in its N-terminus [111], and to TOPBP1 via a conserved phosphorylation site in its C-terminus – Thr202 – which binds specifically to BRCT1 of TOPBP1 with high affinity [80]. The considerably higher affinity for BRCT1 of RHNO1 compared to RAD9 suggests that the former is likely to be the predominant ligand for this site, in a 9–1–1 – RHNO1 – TOPBP1 complex [112]. However, if TOPBP1 does self-associate as has been suggested [89] RHNO1 and RAD9 could cooperate in linking a TOPBP1 dimer to a single DNA-bound 9–1–1 clamp.

### 3.5 Specificity of TOPBP1 BRCT domains

**Fig. 4:**
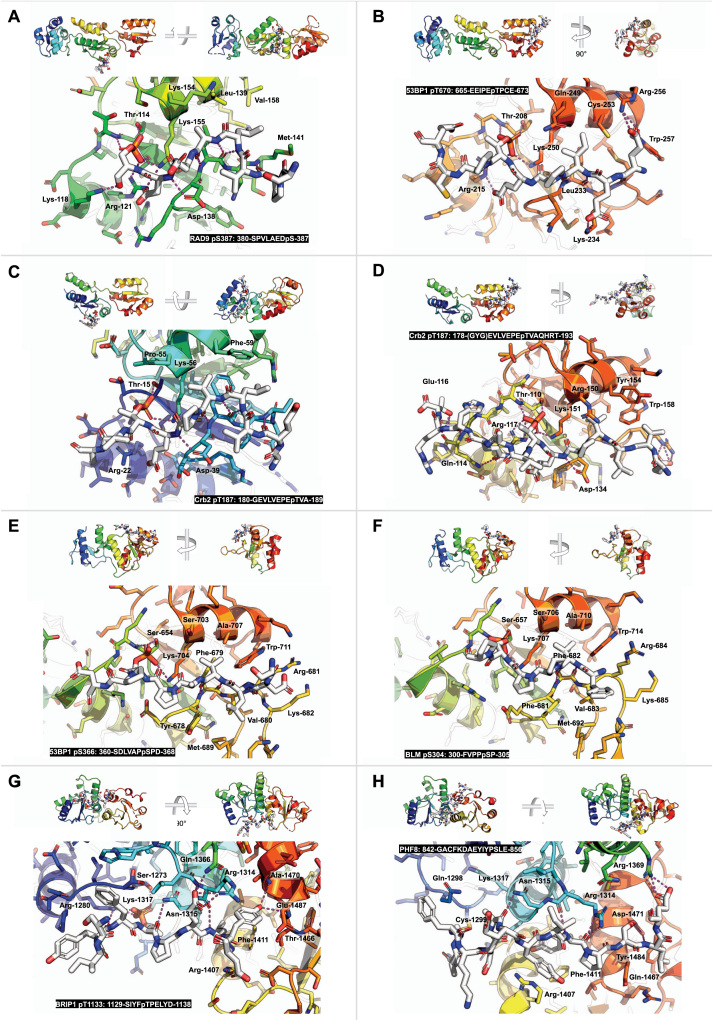
**Peptide bound structures of the BRCT modules of TOPBP1.** BRCT domain secondary structure cartoons are rainbow-coloured (N-terminus blue - > C terminus red), the various peptides shown as white sticks, and with dashed magenta lines indicating hydrogen bonding interactions between the chains. Structural determinants of specificity are described in the text. (**A**) RAD9 pS387 bound to BRCT 1 (PDB: 6HM5) [80]. (**B**) 53BP1 pT670 bound to BRCT 2 (PDB: 6RML) [77]. (**C**) Crb2 pT187 bound to BRCT 1 of Rad4 BRCTs 12 (PDB: 4BU0) [59]. (**D**) Crb2 pT187 bound to BRCT 2 of Rad4 BRCTs 12 (PDB: 4BU1) [59]. (**E**) 53BP1 pS366 bound to BRCTs 45 (PDB: 6RMM) [77]. (**F**) BLM pS304 bound to BRCTs 45 of mouse TOPBP1 (PDB: 5U6K) [63]. (**G**) FANCJ/BACH1 pT1133 bound to BRCTs 78 (PDB: 3AL3) [55]. (**H**) PHF8 bound to BRCTs 78 (PDB: 7CMZ) [68].

The structural and biochemical characterisation of a number of TOPBP1/Rad4 complexes with ligand proteins, provides insight into the sequence specificity of the phosphorylation-dependent interactions made by the competent BRCT domains [80]. Phospho-threonines are the predominant modified residue in binding motifs for both domains, but phospho-serine, as in RAD9-pSer387 and treslin-pSer1001, is certainly not ruled out. Both BRCT1 and BRCT2 display a strong requirement for a small/medium hydrophobic residue − 3 relative to the phosphorylated residue, and Asp/Glu is common in the − 1 position for both (Fig. 3**D)**. Proline is also common at the + 1 position, but the peptide side-chain makes no direct contacts, and its presence is more to do with the specificity requirements of the kinase – often a CDK – that is responsible for the phosphorylation of the motif, rather than its interaction with the BRCT domains. The major distinction in binding preference comes from the presence of a large hydrophobic pocket in BRCT1 that allows binding of two consecutive hydrophobic residues (−2, −3), with a third (−4) folding back against the backbone of the ligand peptide, stabilising a kinked conformation (Fig. 4**A**). In BRCT2, this pocket can only accommodate the − 1 hydrophobic residue, so that the peptide binds in an extended β-sheet conformation that permits a hydrophilic residue at −2 (Fig. 4**B**). Thus, the main selectivity between BRCT1 and BRCT2 results from motifs with hydrophilic residues at − 2 and/or − 3 not being able to bind BRCT1. Motifs with three consecutive hydrophobic residues (−2, −3, −4) that bind BRCT1 (e.g. Crb2-pThr 187), may also be able to bind to BRCT2, but do so with a different conformation (Fig. 4**C and D**).

Only two *bona fide* ligand complexes for the second phospho-binding BRCT module TOPBP1-BRCT4,5 (3,4 in Rad4/Dpb11) have been structurally characterised [63], [77]. These both show a very similar binding conformation for the ligand motif as in BRCT2, with residues − 4 to + 1 in an extended β-sheet conformation, dictated by a comparably small hydrophobic pocket accommodating the hydrophobic side-chain of the − 3 residue. Comparison of these with biochemically identified binding motifs for Rad4/Dpb11 from *S.pombe* Rad9 and *S.cerevisiae* Sld2 (Fig. 3**D)**, reveals an additional strong preference for a proline residue at − 1. In both BLM-pS304 and 53BP1-pS366 complexes with TOPBP1-BRCT4,5, this proline forms a key part of a hydrophobic sandwich, which, along with the main chain of the − 2 and − 3 residues, and the side chain of the − 4 residue, surrounds the protruding side chain of TOPBP1-Tyr678 (Fig. 4**E and F**).

The only phosphorylation-dependent ligand interaction so far characterised for the BRCT7,8 module, which is absent from the yeast TOPBP1-homologues, is with FANCJ/BACH1 [55], [56] (Fig. 3**D)**, and is very similar to complexes of other phosphorylated motifs to BRCT_2_ modules (Fig. 4**G),** although it displays a substantial induced-fit mechanism not evident in other BRCT_2_ modules. Although not phosphorylation dependent, the recently reported interaction of PHF8 involves the same face of BRCT7,8 [68] (see text above and Fig. 4**H**) and would be competitive with binding of phosphorylated ligands to that site.

### 3.6 Combinatorial complexity

The multiple binding sites for different ligands that TOPBP1 provides, underlies its function as a scaffold protein, mediating and reinforcing the co-localisation of proteins that may have little inherent affinity for each other, but can collaborate and co-operate through their association with a common binding partner. Central to this is the phosphorylation independent interaction of the PI3-kinase-like kinase ATR, with the ATR Activating Domain (AAD) of TOPBP1/Rad4/Dpb11, which is necessary for the activation of ATR signalling [103], [104], [105], and appears to be the unique function of this conserved region of TOPBP1/Rad4/Dpb11. Interactions with other ligands via the more promiscuous BRCT domains, then provides a model for coupling ATR activation to whatever DNA context is dictated by any other ligand or ligands bound simultaneously to the same TOPBP1 molecule, or potentially same TOPBP1 oligomer, that binds ATR. While this model holds for TOPBP1 roles in DNA damage signalling, the AAD seems to be dispensable for its role in CMG assembly [93].

## 4 Conclusion

All the multi-BRCT modules of TOPBP1 have been implicated in binding to multiple ligands, and although no direct ligands of BRCT0 or the singleton BRCT domains 3 and 6 have yet been definitively identified, there is no reason to assume that these will be different. This raises the possibility of a substantial number of different TOPBP1-based complexes that could exist, incorporating different combinations of ligands bound to the different attachment points the scaffold provides. If as has been suggested, TOPBP1 molecules self-associate into dimers or even oligomers [81], [87], [88], [89], then we are presented at least in principle, with a huge combinatorial tangle in which TOPBP1 and all its ligands might be simultaneously present. This may be modulated by different phosphorylation-dependent ligands only associating with TOPBP1 at defined cell cycle stages e.g. treslin in S-phase [113] and MDC1 in mitosis [81], or in defined nuclear locations e.g. Treacle/TCOF1 in nucleoli, or following specific signalling events e.g. 53BP1 following DNA damage in G1 [74], [77], but the potential complexity of multi-ligand complexes remains substantial and has been only lightly investigated.

**Fig. 5:**
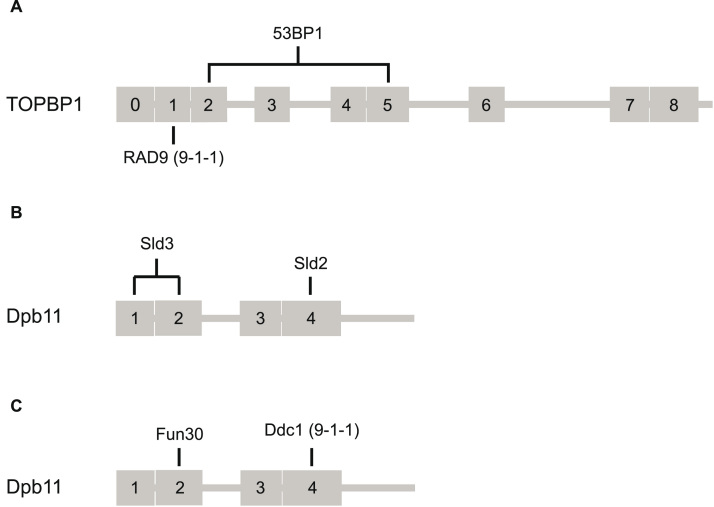
**TOPBP1 multiprotein complexes.** Schematics of experimentally determined ligand combinations compatible with simultaneous interaction with a single TOPBP1 molecule.(**A**) 53BP1 interacts with BRCT domains 2 and 5 while the RAD9 component of 9–1–1 interacts with BRCT1. (**B**) Sld3 binds BRCT domains 1 and 2 of Dpb11, while Sld2 binds BRCT4. A similar arrangement is likely in *S.pombe*. (**C**) Fun30 binds BRCT domain 2 of Dpb11, while the Ddc1 component of the *S.cerevisiae* 9–1–1 complex binds BRCT4. In the equivalent metazoan arrangement, SMARCAD1 – the homologue of Fun30 – also interacts with BRCT2, whereas the RAD9 component of 9–1–1 binds BRCT1.

Simultaneous interaction of two phosphorylation-dependant ligands has been inferred genetically in *S.cerevisiae* for Sld2 and Sld3 binding to Dpb11 [114], and demonstrated directly for the Ddc1 component of yeast 9–1–1 (Ddc1-Mec3-Rad17) and Fun30 binding to Dpb11 [61], and for binding of RAD9 in 9–1–1 and 53BP1 to TOPBP1 [77]. In all three cases, the binding sites for the two ligands are fully compatible with simultaneous binding to a single TOPBP1 molecule. Thus, Sld2 and Ddc1 bind to the BRCT4 module while their respective partners in a ternary complex with Dpb11, Sld3 and Fun30, bind to the BRCT1,2 module. Similarly, RAD9 in human 9–1–1 binds to BRCT1 of TOPBP1, while 53BP1 engages in parallel with BRCT2 and BRCT5. Although not yet experimentally confirmed, the likely interaction of the metazoan homologue of Fun30, SMARCAD1, with BRCT2, would allow a similar co-operation with 9–1–1 (Fig. 5). By contrast, the apparent simultaneous engagement of RAD9 and RHNO1 [89], which both bind selectively to BRCT1 of TOPBP1 [80], can only be explained by interaction with separate TOPBP1 molecules within an oligomeric, or at least dimeric, arrangement.

Since its discovery a quarter of a century ago, TOPBP1 has become one of the central players in genome stability through its pleotropic involvement in DNA replication, damage signalling and repair, but how these different functions interrelate and how they are sustained and reconciled by a single protein, is very poorly understood. Future studies addressing the temporal and spatial distribution of the many complexes TOPBP1 orchestrates, as well as their composition and three-dimensional structure, will provide new insights into this fascinating scaffold protein.

## Declaration of Competing Interest

The authors declare no conflicts of interest.
